# In Silico Simulation of Corticosteroids Effect on an NFkB- Dependent Physicochemical Model of Systemic Inflammation

**DOI:** 10.1371/journal.pone.0004706

**Published:** 2009-03-10

**Authors:** Panagiota T. Foteinou, Steve E. Calvano, Stephen F. Lowry, Ioannis P. Androulakis

**Affiliations:** 1 Biomedical Engineering, Rutgers University, Piscataway, New Jersey, United States of America; 2 Department of Surgery, UMDNJ-Robert Wood Johnson Medical School, New Brunswick, New Jersey, United States of America; Fondazione Telethon, Italy

## Abstract

**Background:**

During the onset of an inflammatory response signaling pathways are activated for “translating” extracellular signals into intracellular responses converging to the activation of nuclear factor (NF)-kB, a central transcription factor in driving the inflammatory response. An inadequate control of its transcriptional activity is associated with the culmination of a hyper-inflammatory response making it a desired therapeutic target. Predicated upon the nature of the response, a systems level analysis might provide rational leads for the development of strategies that promote the resolution of the response.

**Methodology and Findings:**

A physicochemical host response model is proposed to integrate biological information in the form of kinetic rules and signaling cascades with pharmacokinetic models of drug action for the modulation of the response. The unifying hypothesis is that the response is triggered by the activation of the NFkB signaling module and corticosteroids serve as a template for assessing anti-inflammatory strategies. The proposed *in silico* model is evaluated through its ability to predict and modulate uncontrolled responses. The pre-exposure of the system to hypercortisolemia, i.e. 6 hr before or simultaneously with the infectious challenge “reprograms” the dynamics of the host towards a balanced inflammatory response. However, if such an intervention occurs long before the inflammatory insult a symptomatic effect is observed instead of a protective relief while a steroid infusion after inducing inflammation requires much higher drug doses.

**Conclusions and Significance:**

We propose a reversed engineered inflammation model that seeks to describe how the system responds to a multitude of external signals. Timing of intervention and dosage regimes appears to be key determinants for the protective or symptomatic effect of exogenous corticosteroids. Such results lie in qualitative agreement with *in vivo* human studies exposed both to LPS and corticosteroids under various time intervals thus improving our understanding of how interacting modules generate a behavior.

## Introduction

The systemic inflammatory response syndrome (SIRS) often accompanies critical illnesses and can be an important cause of morbidity and mortality [1]. It is evoked by many stimuli including infection, trauma, invasive surgery and biological stressors in general and is characterized by a cascade of events during which multiple cell types are deployed to locate pathogens, recruit cells and eventually eliminate the offenders and restore homeostasis. Under normal circumstances, the dynamics of an acute inflammatory response are tightly regulated [2]; however when anti-inflammatory processes fail an amplified inflammation can turn what is normally a beneficial reparative process into a detrimental physiological state which is characterized by severe, uncontrolled systemic inflammation and multiple organ dysfunction [3].

Despite our growing understanding of the cellular and molecular mechanisms of SIRS [4] and the success of pre-clinical studies, not many effective therapies exist [5], [6], [7], [8], [9], [10], [11], [12], [13]. A key reason for this conundrum is the difficulty in predicting how the complex dynamics of inflammation are modulated. Since successful interventions depend on the stage and trajectory of the response, systems-oriented approaches have been advocated for the control of physiological responses [14]. Thus, significant opportunities emerge in the context of systems biology which aims at the deconvolution of complex phenomena, such as the inflammatory response, to their constitutive elements and the quantification of the dynamic interactions among these elements through appropriate computational models. Mathematical models integrating the interacting elements of the inflammatory response offer the opportunity to establish causal relationships and evaluate putative intervention strategies [15].

A number of excellent prior studies [16], [17], [18], [19], [20], [21] have placed emphasis on simulating inflammation based on the kinetics of well-defined markers [22], [23]. The key characteristic of these models is the *a priori* postulation of specific components (cytokines etc.) that are consistent with prior biological knowledge. Appropriate interactions between components and their associated dynamics are subsequently evaluated. In an attempt to integrate high-throughput transcriptional data we recently introduced a systems level approach [24], [25] that decomposes high-dimensional microarray data into a critical set of dynamic features that are considered to be the elementary inflammatory responses triggered by an endotoxin stimulus in peripheral blood leukocytes (PBLs). Our fundamental assumption is that the transcriptional signatures capture the cellular dynamics in response to the inflammatory agent. These constitutive dynamics features are considered to be the “blueprints” of the orchestrated dynamics of the perturbed biological system and in order to study the underlying complexity of an *in vivo* human response to endotoxin a semi-mechanistic indirect response model was proposed in [26]. Our approach couples receptor mediated phenomena with transcriptional effects based on ligand-receptor kinetics. One of the key assumptions underpinning our prior modeling effort is that intracellular signaling cascades activating inflammation-specific transcriptional responses can be mathematically approximated by an aggregate variable serving as a proxy of the activating signal. However, during the onset of an inflammatory response signaling pathways are activated for “translating” extracellular signals into intracellular responses [27]. Such a signal transduction cascade converges to the activation of effector proteins (transcription factors) that regulate the expression of critical genes. Therefore, understanding more about the complex inflammatory reactions would require the development of computational models that incorporate biological information in the form of critical signaling cascades and kinetic rules. We wish therefore to deconvolute and interpret the combined activating signal with its “mechanistic” equivalence developing more interpretable and biologically relevant systems based models of inflammation.

The work to be discussed in this paper aims to address the possibility of a semi-mechanistic host response model that integrates signaling and pharmacokinetic models of drug action for the modulation of the inflammatory response. We opt therefore to develop a reverse-engineered model of endotoxin-induced human inflammation that couples elementary signaling pathways with pharmacokinetic models of corticosteroids, as putative controllers of the inflammatory response. Nuclear factor (NF)-kB is a central transcription factor that plays a major role in driving the inflammatory response [28]. We test the hypothesis that the activation of NFkB signaling module serves as the representative signaling controller of the pro-inflammatory genetic switch underpinning the manifestation of transcriptional responses. An inadequate control of its transcriptional activity is associated with the culmination of a hyperinflammatory response making it a desired therapeutic target. Anti-inflammatory drugs such as corticosteroids play a critical role in modulating the progression of inflammation interfering either transcriptionally with the activity of NF-kB [29], [30] or priming anti-inflammatory cytokines such as IL-10 [31]. Effectively, the dynamic integration of regulatory signaling information with the anti-inflammatory effect of corticosteroids, , sheds some light on how the system responds to a multitude of external signals offering the possibility of performing *in silico* experiments that would eventually allow us to rationalize the success/failure of particular interventions. It is the ultimate goal of this study to trace the non-linear inflammatory signal more efficiently thus improving our understanding of how interacting modules respond to generate a behavior. The proposed integrated model of systemic inflammation prior to any intervention is characterized by the dynamic state of eleven (11) variables that describe the propagation of LPS signaling through interacting modules. Its evaluation is demonstrated through a series of biologically relevant scenarios indicative of the non-linear dynamics of inflammation. These scenarios involve the implications of increased host susceptibility to endotoxin stimulus followed by systematic perturbations in the regulatory signaling module. Simulating the trajectory of an unconstrained inflammatory response allows us to perform computational tests for the therapeutic evaluation of corticosteroid based intervention strategies. The corticosteroid intervention envelope consists of five (5) deterministic equations that take drug binding interactions into account seeking to describe the elementary reactions of the cellular signaling of steroids. The pre-exposure of the system to hypercortisolemia, i.e. 6 hr before or simultaneously with the main endotoxin challenge “reprograms” the intrinsic dynamics of the host towards a balanced (suppressed) inflammatory response. However, if such an intervention occurs long before LPS (i.e. 12 hr or 144 hr) a symptomatic effect is observed instead of a protective relief while a steroid infusion after inducing inflammation requires much higher drug doses. Therefore, timing of intervention and dosage regimes appears to be key determinants for the protective or symptomatic effect of exogenous corticosteroids on the progression of inflammation. Qualitatively, such *in silico* results lie in agreement with *in vivo* human studies exposed both to LPS and corticosteroids under various time intervals thus paving the way for improving the working feedback loop between “dry” and “wet” experiments.

## Results

### Qualitative assessment of NFkB-dependent indirect response model of systemic inflammation

**Figure 1:**
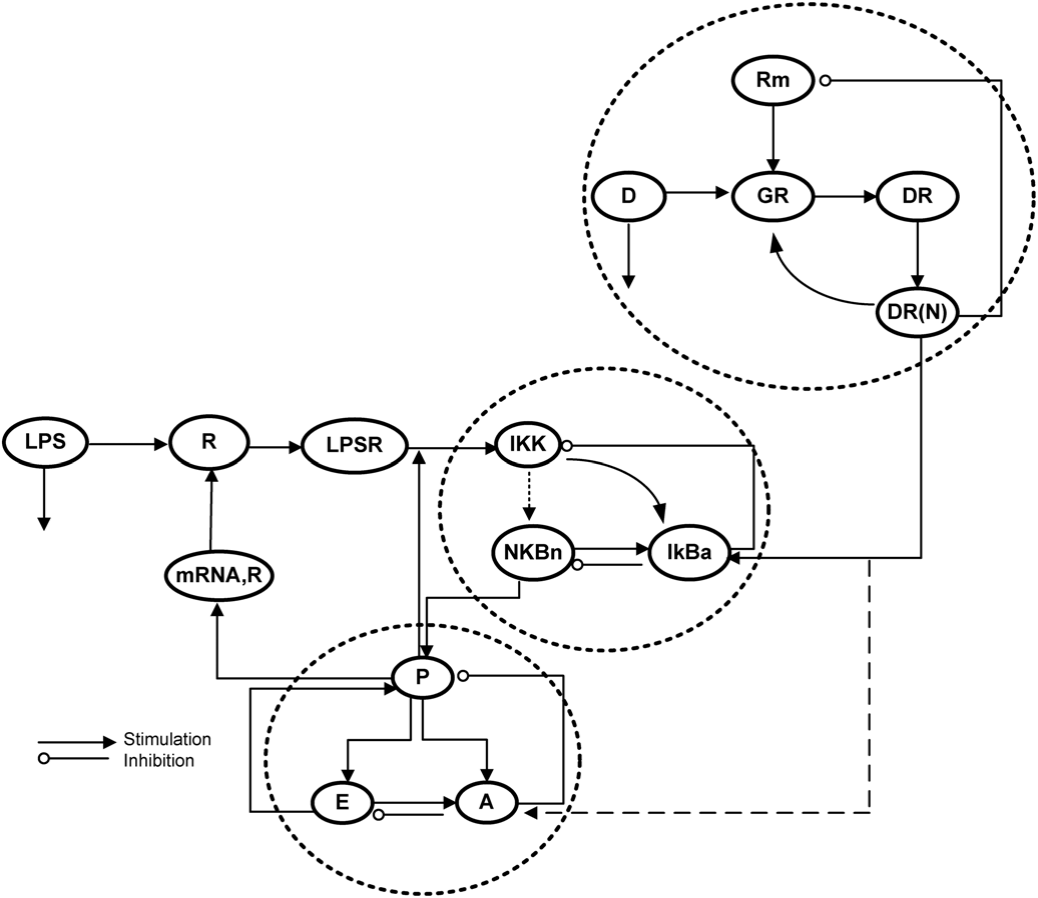
Schematic illustration of a reverse engineered model of systemic inflammation. Interacting modules involve the propagation of LPS signaling on the transcriptional response level coupled with the anti-inflammatory effect of corticosteroids. The propagation of LPS signaling involves the interaction of the inflammatory stimulus, LPS with its receptor (R) forming the surface complex (LPSR) which activates IKK activity. The IKK-dependent signal activates the translocation of NF-kB (NFkB_n_) through phosphorylation and degradation of its primary inhibitor, IkBa. The nuclear NFkB (NFkBn) is auto-regulated by its inhibitor protein, IKBa and stimulates the production rate of the pro-inflammatory response (P) while there is certain connectivity among the essential transcriptional signatures (P, A, E). The mRNA of the receptor (mRNA,_R_) is stimulated by pro-inflammation (P) and it is translated to the surface protein (R). The corticosteroid intervention envelope consists of the corticosteroid drug (D) which binds to its intracellular receptor (GR) forming the cytosolic complex (DR) that translocates to the nucleus (DR(N)) and modulates the dynamics of inflammation via an upregulation of anti-inflammatory proteins (IkBa, A). The nuclear complex (DR(N)) auto-regulates the transcription of its receptor (GR) and a portion of nuclear receptor DR(N) is recycled. The potentiating effect of DR(N) to A is represented by dashed lines as *in silico* results refer to corticosteroid perturbations on IKBa. Qualitatively, similar results are observed if the mode of action involves upregulation of the anti-inflammatory response (A).

We have previously demonstrated that the transcriptional dynamics of human leukocytes exposed to bacterial endotoxin can be decomposed into to three elementary comprehensive responses [24], [26]. Unlike previous approaches that concentrate on specific biomarkers, these elementary responses capture the functional dynamics and were shown to be related to pro-inflammatory (P), anti-inflammatory (A) and energetic (E) transcriptional events associated with the overall host response. The response is triggered by the activation of the NFkB signaling module as a result of the formation of an activating signal associated with the binding of LPS to appropriate receptors. We hypothesize that NFkB serves as a proxy for the inflammation specific transcription factors that initiates the expression of pro-inflammatory genes while its activity is primarily modulated by the kinase activity (IKK) and the inhibitor (IKBa). In this study, we seek to describe the host response to endotoxin via interacting modules that involve the propagation of LPS signaling on the transcriptional response level through NFkB dependent mechanism and the genomic signaling of exogenous corticosteroids, as the putative controllers of inflammation. The corticosteroid intervention envelope consists of a set of elementary interactions that involve: (i) the binding of the corticosteroid drug (D) to its cytosolic receptor (GR), (ii) the subsequent formation of the drug-receptor complex (DR) (iii) the translocation of the cytosolic complex to the nucleus (DR(N)) that alters the transcriptional machinery activating or repressing numerous genes and finally (iv) the autoregulation of the gene transcript of the glucocorticoid receptor (R_m_). All the interacting components and modules that constitute the NFkB dependent physicochemical model of inflammation are shown in Figure 1.

**Figure 2:**
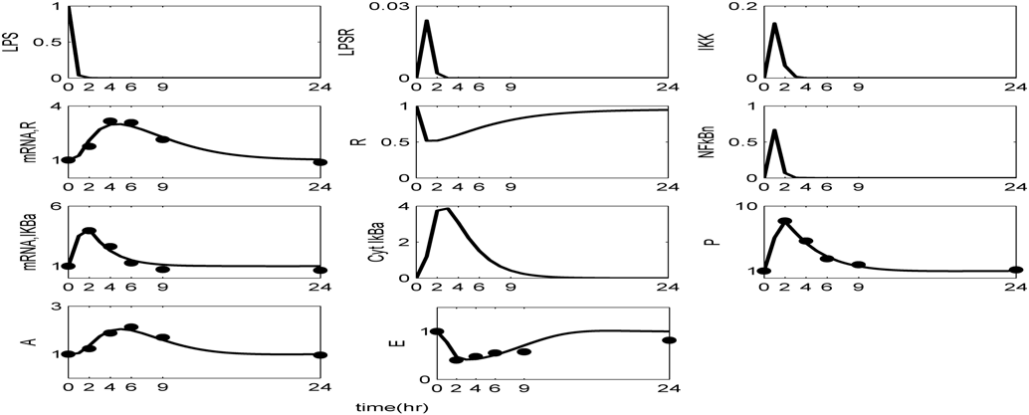
Estimation of relevant model parameters. Temporal profiles of the elements that constitute the NFkB dependent model of endotoxin-induced inflammation. Solid lines (-) correspond to model predictions whilst the symbols (•) denote for the experimentally measured transcriptional signatures.

**Figure 3:**
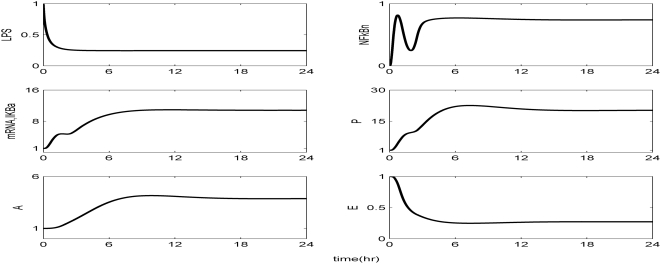
Temporal responses of model elements in a persistent infectious inflammatory response. Reducing the degradation rate of LPS to half of its initial value we simulate the case of an unsuccessful clearance of LPS that accounts for the sustained (aberrant) activity of NFkB leading to a chronic inflammatory response.

**Figure 4:**
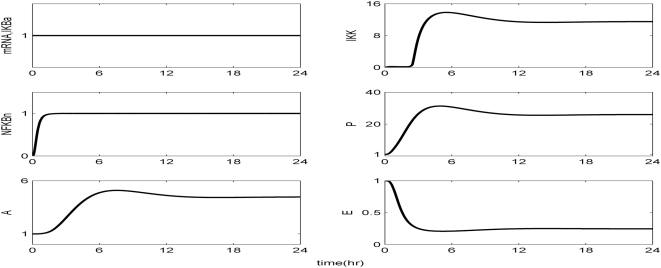
Simulation of a knock-out *in silico* experiment (IkBa^−/−^). Manipulating the model so that there is no *de novo* transcriptional synthesis of NF-kB inhibitor (IkBa) which is responsible for the absence of NF-kB auto-regulatory feedback loop. Such a scenario accounts for maladapted activity of NFkBn that triggers an uncompensated inflammatory response.

**Figure 5:**
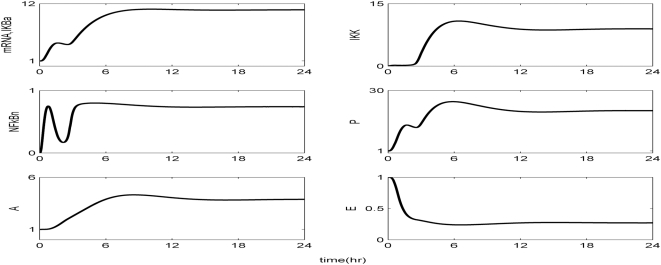
Pre-existence of pro-inflammatory mediators may enhance abnormally the intracellular signaling through IKK. Such a response leads to an unconstrained activity of NFkBn that drives downstream a persistent pro-inflammatory response which cannot be counter-regulated by the anti-inflammatory arm of the host defense system. Such a mode of dysregulation is simulated by manipulating the zero production rate of pro-inflammation (Kin,P) so that Kin,P(unhealthy response) ∼2* Kin,P (healthy response).

Kinetic parameters are estimated in order to best reproduce the essential transcriptional responses associated with experimental measurements, (Table 1). The reconstructed dynamic profiles associated with a self-limited inflammatory response, of the major transcriptional signatures coupled with the elementary signaling molecules of NF-kB pathway are presented in Figure 2. In essence, a self-limited inflammatory response involves the successful elimination of the inflammatory stimulus within the first 2 hr post-endotoxin administration while followed by a subsequent resolution within 24 hr. We assess the appropriateness of the structure of the proposed model by simulating a malfunction in the clearance rate of pathogen-derived endotoxin, Figure 3. Such a case is simulated by manipulating (decreasing) the parameter associated with the degradation rate of LPS, k_LPS,2_. Although decreased degradation of LPS is not associated with a defined clinical condition it is possible that this phenomenon may exist. For example, it is known that triglyceride-rich lipoproteins bind to LPS and that these complexes are cleared by binding to lipoprotein receptors. Furthermore, these receptors are abundant in the liver which clears ∼70% of lipoproteins from the circulation. Therefore, it can be postulated that patients with liver dysfunction may have impaired clearance of LPS. As shown in Figure 3 the inflammatory stimulus persists and leads to an aberrant NFkB activity that drives downstream a chronic inflammatory response. We further evaluate the proposed *in silico* model by exploring the possibility of a mechanistic maladaption in the dynamics of the regulatory NFkB signaling module. As illustrated in Figure 4, performing an *in silico* IkBa^−/−^ knock-out experiment we simulate a sustained inflammatory response that fails to resolve. Another mode of perturbation of the underlying dynamics of the probed system is related to the presence of a “prior” insult that coupled with the LPS stimulus account for an overwhelming production of pro-inflammatory mediators, Figure 5. Such a sustained pro-inflammatory signaling deregulates the NFkB signaling module leading to a persistent NFkB activity. Such persistence implies that the nuclear concentration of NFkB cannot be further constrained by its primary inhibitor, IkBa and eventually settle to a steady state far away from their equilibrium (homeostasis). We simulate such a scenario by manipulating the zero order production rate of the pro-inflammatory response (K_in,P_) and particularly increasing it twice its initial value.

**Table 1:** Estimated values of the parameters based on self-limited response data.

Parameter	Value	Parameter	Value	Parameter	Value	Parameter	Value
**k** ***_LPS_***_**,1**_	4.500	k_in,mRNA,R_	0.090	k_I,2_	0.870	k_P,R_	1.740
**k** ***_LPS_***_**,2**_	6.790	k_out,mRNA,R_	0.250	K_in,P_	0.030	k_P,1_	29.740
**k_syn_**	0.020	k_NFkB,1_	16.290	K_out,P_	0.330	k_P,2_	9.050
**k_1_**	3.000	k_NFkB,2_	1.180	K_in,A_	0.090	k_A,1_	0.010
**k_2_**	0.040	K_in,IKBa_	0.460	K_out,A_	0.590	k_A,E_	5.300
**k_3_**	5.000	k_IkBa,1_	13.270	K_in,E_	0.080	k_E,P_	2.210
**k_4_**	2.240	k_I,1_	1.400	K_out,E_	0.280		

The values for k_1_ and k_2_ are taken from [75].

### Modulating the progression of an unresolved inflammatory response

**Figure 6:**
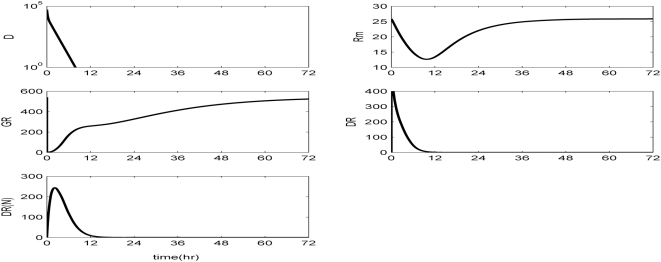
Dynamic evolution of model elements that constitute the mechanistic pharmacokinetic model of corticosteroids action given the parameters and initial conditions extracted from [52].

**Figure 7:**
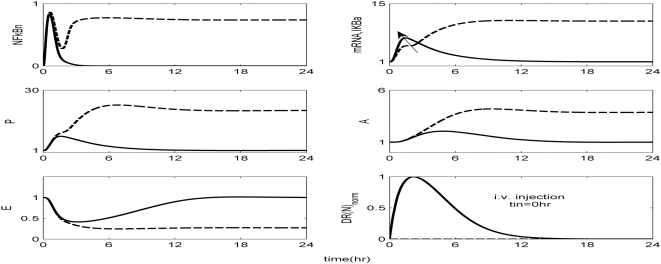
Exploring the mode of corticosteroid action in enhancing the transcriptional synthesis of IkBa which is illustrated by the solid arrow. An i.v. injection of the corticosteroid drug administered concomitantly with endotoxin (t_in_ = 0 hr) suffices to reverse (prevent) the lethal effect of a high dose of endotoxin. Solid lines (-) correspond to the inflammatory resolution due to the corticosteroid infusion at t = 0 hr while dashed lines (--) simulate the progression of inflammation in response to a high concentration of LPS (i.e. LPS(t = 0 hr) = 4).

**Figure 8:**
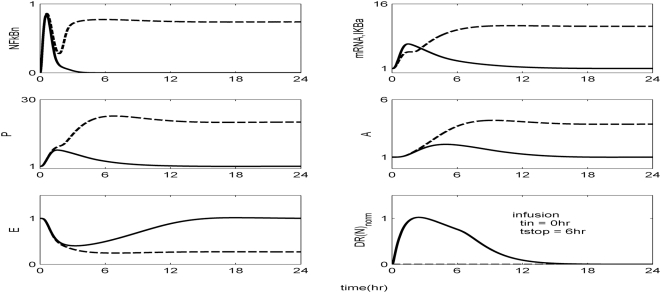
Exploring the effect of corticosteroids on CORT-LPS group. The drug is administered as a continuous infusion initiated simultaneously with LPS administration (t_in_ = 0 hr) for 6 hr (t_stop_ = 6 hr) and we observe a resolution in the progression of inflammation.

**Figure 9:**
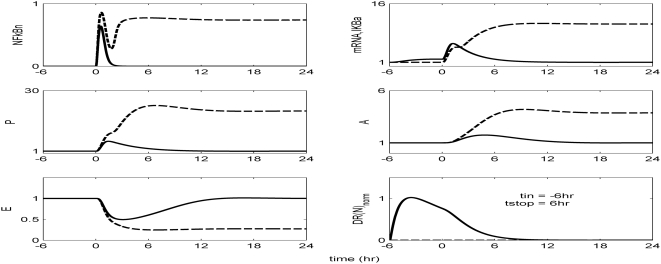
Hypercortisolemia for 6 hr prior to LPS challenge (t_in_ = −6 hr). The system is pre-exposed for 6 hr to a continuous infusion of corticosteroids while it is continued for another 6 hr after the endotoxin challenge (t_stop_ = 6 hr). Such an intervention “reprograms” the dynamics of the system modulating the effect of a high LPS concentration.

**Figure 10:**
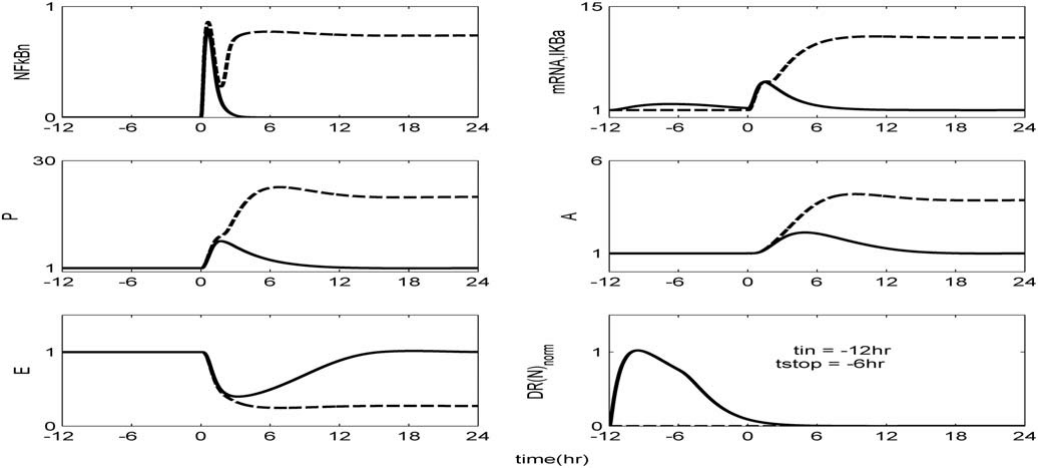
Exploring the effect of a continuous infusion of corticosteroids for 6 hr initiated at 12 hr prior to LPS (t_in_ = −12 hr) and elapsed at t = −6 hr before the administration of the inflammatory stimulus (LPS), (CORT-6-LPS). Such hypercortisolemia modulates significantly the progression of a systemic inflammatory response syndrome.

**Figure 11:**
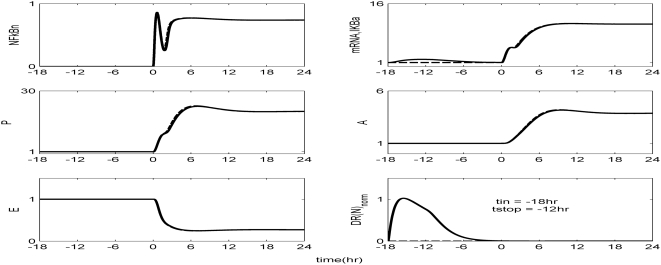
Pre-exposure the system into hypercortisolemia which is initiated as a continuous infusion 18 hr before the endotoxin challenge (t_in_ = −18 hr) and continued for 6 hr (t_stop_ = −12 hr), (CORT-12-LPS). Such intervention strategy does not have a profound effect in the dynamic state of the system while the progression of an unresolved inflammation (solid lines) continues after the termination of steroid infusion.

**Figure 12:**
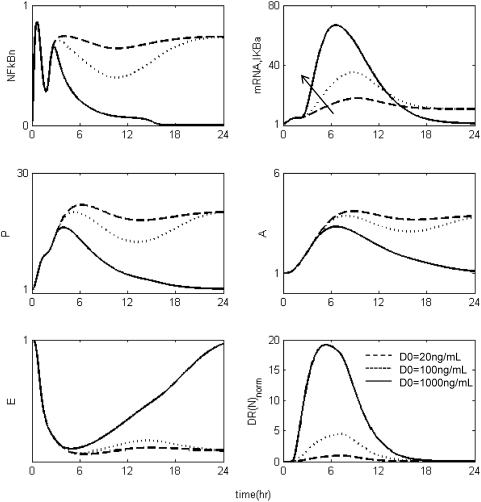
Dose-dependent modulation in the progression of the inflammatory response due to corticosteroids initiating infusion at t = 1 hr and for 6 hr post-endotoxin administration at multiple doses (D0). The solid arrow illustrates the mode of corticosteroid action via up-regulation of mRNA,IkBa. Solid lines (-) characterize a resolution in the inflammatory response while dashed lines (--) and dotted (…) correspond to lower drug doses that does not regulate properly the aberrant activity of NFkB if the intervention is initiated after the endotoxin challenge. The DR(N) profile that corresponds to the lower drug dose, D0 = 20 ng/mL, constitutes the basis active signal normalized to (0,1) values, while the active signals for larger doses are scaled with respect to the lowest drug dose.

**Figure 13:**
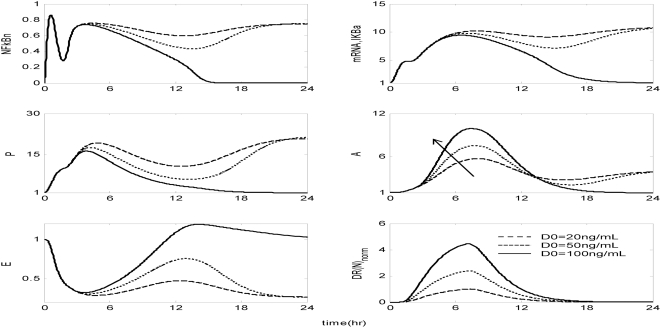
Explore the effect of corticosteroids at multiple drug doses initiated at t = 1 hr and continued for 6 hr after the endotoxin challenge priming the production rate of IL10 signaling (A component). The effect of corticosteroids towards A signaling is illustrated by the solid arrow. Solid lines characterize a resolution in the progression of systemic inflammation whereas dashed and dotted lines correspond to lower drug doses that cannot sufficiently reverse the progression rate of an aberrant inflammation. All the active signals, DR(N)norm, have been normalized with respect to the lowest drug dose, D0 = 20 ng/mL.

The *in silico* model of inflammation enables us to predict an inflammatory response that does not properly abate making it a critical enabler for the evaluation of corticosteroid-based intervention strategies. Characteristic dynamics of the profiles of the signaling molecules that constitute the corticosteroid intervention envelope are presented in Figure 6. An intravenous injection of the drug, via the activation of intermediate signaling steps, eventually leads to the up-regulation of the active complex, DR(N). Based on the mode of corticosteroids action defined, to be discussed in detailed in the Methods section, we explore the potential of the active signal, DR(N)_norm_, in modulating the progression of an unresolved inflammation, Figure 7. We observe that such a signal mediates the corticosteroid effect on the transcriptional response level primes the dynamic state of NFkB inhibitor so that it suffices to promote resolution of the inflammatory response. Despite the high initial LPS concentration which perturbs the dynamics of inflammation (dashed lines), the corticosteroid intervention in the form of an intravenous (i.v.) injection initiated at t = 0 hr “reprograms” the dynamic state of the system in favor of a balanced regulation (solid lines). While comparing the dashed and solid lines in Figure 7 we observe that the intervention strategy plays a critical role in the dynamics of IkBa during the first 4 hrs post-LPS where suffices to control the intrinsic inflammmatory dynamics favoring homeostasis within 24 hrs. On the other hand, prior to any intervention the system seems to have lost any potential for attenuation and its inability to adapt to high LPS concentration is mathematically translated into unconstrained responses (dashed lines). Therefore, the intervention envelope based on corticosteroids serves as a critical enabler to explore the capability of different intervention strategies in modulating the progression of systemic inflammation. Another illustration of the protective effect of corticosteroids is shown in Figure 8. We simulate a continuous infusion of the steroid drug that is initiated at t = 0 hr (simultaneously with LPS) and continues for 6 hr post-LPS administration (CORT-LPS strategy). As seen in Figure 8 such treatment strategy suffices to reverse the deleterious outcome of a persistent non-infectious inflammatory response (high initial LPS concentration). Moreover, pre-exposing the system before endotoxin challenge for 6 hr to hypercortisolemia we observe a proper modulation on the progression of the inflammatory response as well, Figure 9. The corticosteroid intervention occurs at t = −6 hr followed by the concomitant administration of the endotoxin stimulus at t = 0 hr and the infusion continues for 6 hr after the endotoxin challenge. In addition, similar responses are observed for the system if it is pre-exposed to hypercortisolemia for 6 hr but the steroid intervention is initiated at t = −12 hr (CORT-6-LPS), Figure 10. However, if the system is pre-exposed to hypercortisolemia for the same duration as previously mentioned (6 hr) but the time interval between the termination of infusion and LPS administration is greater (12 h), Figure 11, we observe a blunted effect of the corticosteroid treatment on the progression of inflammation (Cort-12-LPS). Similar results are obtained if the system is exposed to a continuous infusion of hypercortisolemia initiated at t>0 hr after the administration of endotoxin (i.e. t = 1 hr), dashed lines in Figure 12. On the other hand, as shown in Figure 12 the progression of the inflammatory response is differently perturbed on a dose-dependent manner (dashed lines versus solid). Preserving the route of drug administration the active signal DR(N) must increase in magnitude in order for the system to respond to a multitude of external signals (LPS, Drug).Therefore, dose-dependent profiles are simulated in Figure 12 and Figure 13 where there exists a dosage regime that modulates the dynamics of the system towards resolution.

## Discussion

### Malfunction in LPS's clearance rate

The dynamics of the inflammatory response are highly complex such that a maladaption in the homeostasis of the system can be attributed to various reasons. One such possibility is associated with a malfunction in endotoxin clearance rate which corresponds to a higher exposure of the host to the stimulus. In Figure 3 we simulate the case of a persistent inflammatory response which corresponds to an increased exposure of the host response to the inflammatory stimulus (LPS). As shown in Figure 3, the inferred NFkB activity can be characterized as a “two-wave” response; initially it increases due to the inflammatory stimulus while trying to adapt its regulatory activity at 2–3 hr post-endotoxin administration. However, at t>3 hr the activity of NFkB cannot be regulated successfully and it settles to a sustained elevated state that drives downstream the over-excitation of both pro- and anti-inflammatory mediators; leading to an unconstrained inflammatory response. Interestingly, in [32] Klinke et al. aim at exploring experimentally the possibility of modulating the temporal control of NF-kB activation. Macrophages are exposed to a persistent inflammatory stimulus (LPS) and the available experimental data show the presence of a “damped” oscillatory behavior in NF-kB activity.

### Maladaption in the dynamics of NFkB signaling module

The protein inhibitor of NF-kB (IkBa) aims at retrieving nuclear concentration of NF-kB with the formation of an inactive complex in the cytoplasm regulating the expression of various inflammatory genes. The transcription factor NF-kB up-regulates the gene transcript of IkBa (mRNA,_IkBa_) so that the translated protein IkBa serves as the major component for regulating its transcriptional activity.. Thus, in Figure 4 we simulate the case of no transcriptional activity of NF-kB in the promoter region of IkBa. In the absence of NF-kB inhibitor (IkBa^−/−^) there is an aberrant regulatory activity of NF-kB that leads to its persistent nuclear activity driving an inflammatory response that fails to restore homeostasis. Such an *in silico* result has been experimentally tested annotating the impact of such a knock out in inducing a chronic inflammatory response [33].

Additionally a pre-existence of pro-inflammatory cytokines due to the presence of a prior “insult” may deregulate the intracellular dynamics responsible for an amplification of the inflammatory response, Figure 5. In our model such a scenario can be simulated due to the positive feedback interaction between the intracellular critical node (IKK activity) and the pro-inflammatory response that disturbs the bistable behavior of the system. Therefore we attempt to manipulate (increase) the zero order production rate of the essential pro-inflammatory signaling. Clinically, such an increased rate in the production of pro-inflammatory mediators might be the outcome of a surgical trauma followed by bacterial infection, a so called two hit scenario [34].

### Evaluating the corticosteroid intervention envelope in modulating the progression of systemic inflammation

Due to the physiological role of corticosteroids in the immune system [29] researchers have put significant effort in understanding the cytokine dynamics under hypercortisolemia [35], [36], [37], [38], [39], [40]. These studies have focused on elucidating the *in vivo* responses to endotoxin (LPS) when there is an exposure of subjects to hypercortisolemia for various durations of time. Thus, in [35] normal human subjects were exposed to glucocorticoid infusion concurrent with and before the endotoxin challenge. The hydrocortisone infusion lasted for a 6 hr period with subsequent intervening periods of 6 (CORT-6-LPS), 12 (CORT-12-LPS) and 144 hr (CORT-144-LPS) before endotoxin administration or simultaneously with LPS challenge (CORT-LPS). Experimental measurements of cytokines and hemodynamic parameters suggest the integral role of hypercortisolemia in CORT-LPS and CORT-6-LPS groups in modulating the cytokine network characterized by decreased plasma concentrations of various cytokines, i.e. TNF, IL-6 when compared to the group that received only LPS. However, in CORT-12-LPS and CORT-144-LPS the plasma concentrations of the aforementioned inflammatory mediators were significantly increased compared to CORT-LPS and CORT-6-LPS groups. Therefore, such evidence suggest the critical impact of the duration of the corticosteroid intervention before inducing inflammation in perturbing the dynamics of both hormonal and cytokine level.

Herein, we explore the capability of corticosteroids to modulate the inflammatory response under various treatment schedules. As shown in Figure 7 a single i.v. injection of corticosteroids at t = 0 hr suffices to reverse the dynamics in response to the high concentration of LPS. Similar results were obtained if we preserved the timing of intervention but modified the route of drug administration switching to a continuous infusion, Figure 8. Moreover, if the system is pre-exposed to hypercortisolemia for 6 hr and concomitantly with LPS the hypercortisolemia is continued for another 6 hr, Figure 9, the intrinsic dynamics of the system were effectively modulated as well. Such results support an early intervention strategy that targets the regulatory arms of systemic inflammation and successfully capture the dynamic behavior of the system in CORT-LPS and CORT-6-LPS groups of the aforementioned experimental study. Complementary to this, in [41] there is emphasis on the potential of a preoperative administration of corticosteroids in alleviating surgical stress. The underlying hypothesis of such a preoperative exposure stems from the fact that a modification of the inflammatory dynamics at an early stage (transcriptional level) would seem to be beneficial in balancing the immune response given that these anti-inflammatory drugs (corticosteroids) inhibit pro-inflammatory transcription factors (NF-kB).

However, as previously stated, if the corticosteroid intervention is terminated 12 h or 6 days before the administration of LPS the dynamics of the cytokine network are quite different. In order to simulate such a scenario we explore the potential of a continuous infusion of steroids that is terminated 12 h before the endotoxin challenge. As seen in Figure 11 such an intervention strategy fails to reverse the effects of a high concentration of LPS. Similar results are observed if the intervention strategy elapses at times greater than 12 hr from LPS administration (*data not shown here*). The primary reason for such a failure stems from the fact that at t>9 hr the transcriptional profile of IkBa is resolved. Therefore any pre-exposure to corticosteroid infusion that is terminated at t>9 hr would not “reprogram” the inflammatory dynamics towards a reversal in the progression rate of an inflammatory response. In addition to this, exploring the response of the system at later stages of the progression of the inflammatory response, Figure 12, the active steroid signal, DR(N)_norm_, has to increase in magnitude in order for the inflammatory response to be tightly regulated. These *in silico* results lie in agreement with studies [31] that suggest a dose-dependent decrease in LPS-induced TNF in peripheral human blood leukocytes that are exposed to hydrocortisone infusion. A discrepancy between the experimental evidence in [31] and our *in silico* results is related to the concentration of the anti-inflammatory cytokine IL10. In particular, van der Poll and Lowry [31] demonstrate increased plasma concentrations of IL10 at higher corticosteroid doses. In our model, due to our hypothesis that the drug stimulates the transcription rate of IkBa, it is expected to observe the solid trajectories of Figure 12 which simulate decreased inflammatory responses (P, A, E). If we assume that corticosteroids instead of up-regulating the inhibitor of NFkB they prime the production rate of IL10 signaling (A), Figure 12 is extended to Figure 13. As shown in Figure 13 the gradual increase in the anti-inflammatory (A) signaling as the drug dose increases modulates the response of the system towards a more balanced inflammatory response. In addition, the computational experiments presented in Figure 7–Figure 11 can be reproduced if we consider that the corticosteroid intervention envelope perturbs the state of the anti-inflammatory (A) signaling which lie in agreement with the pleiotropic mode of corticosteroids anti-inflammatory activity. Despite the controversies regarding the administration of either high-dose steroids for the short-term in septic patients [42] or the prolonged use of low dose steroids in clinical settings [43] the present study provides qualitative insight on how the system responds to various intervention strategies opening challenging windows towards the design of effective drug treatment schedules [44].

In summary we have developed a semi-mechanistic host response model that describes the dynamic evolution of an *in vivo* human response to endotoxin. Interacting components involve elementary signaling pathways that propagate extracellular signals to the transcriptional response level and pharmacokinetic models of corticosteroids, as putative controllers of the inflammatory response. Model parameters are appropriately evaluated so that to reproduce a self-limited inflammatory response that resolves within 24 hr post-endotoxin administration. The potential of the model is demonstrated via computational tests performed to reproduce biologically relevant scenarios associated with an increase in host's susceptibility to endotoxin stimulus as well as in the regulatory interactions of signaling cascades. Exploring the possible effects of systemic perturbations enables us to trace the dynamics of a systemic inflammatory response syndrome. *In silico* experiments that activate the corticosteroid intervention envelope in order to modulate the progression of inflammation, encourage the proper design of intervention strategies that target early arms of the host response modulating the activity of crucial pro-inflammatory transcription factors. Such a modeling framework can potentially offer significant insight as to how a host undergoing an inflammatory response responds to a multitude of external signals through interacting signaling modules and possible strategies for restoring homeostasis. The work discussed in this study lays the foundation for an *in silico* “disease” progression model that sheds light on the nature of disease and how it responds to pharmacological interventions which is central to translational systems biology [45], [46].

## Materials and Methods

### Human endotoxin model and data collection

The data used in this study were generated as part of the Inflammation and Host Response to Injury Large Scale Collaborative Project funded by the USPHS, U54 GM621119 [47], [48]. Human subjects were injected intravenously with endotoxin (CC-RE, lot 2) at a dose of 2-ng/kg body weight (endotoxin treated subjects) or 0.9% sodium chloride (placebo treated subjects). Following lysis of erythrocytes and isolation of total RNA from leukocyte pellets, [47], biotin-labeled cRNA was hybridized to the Hu133A and Hu133B arrays containing a total of 44,924 probes for measuring the expression level of genes that can be either activated or repressed in response to endotoxin. A set of 5,093 probe sets were characterized by significant variation (corresponding to 0.1% false discovery rate) across the time course of the experiment using the SAM software [49]. The data are publicly available through the GEO Omnibus Database (http://www.ncbi.nlm.nih.gov/geo/) under the accession number GSE3284. The data have been appropriately de-identified, and appropriate IRB approval and informed, written consent were obtained by the glue grant investigators [47].

### An indirect response model of human endotoxin-induced inflammation

In the proposed model, the inflammatory response is activated when endotoxin is recognized by pathogen recognition receptors [50]. Such recognition process involves the induction of a signal transduction cascade that triggers downstream critical signaling modules for the activation of transcriptional factors that play a critical role for the transcriptional initiation of inflammatory genes. Our inability to precisely model such a cascade of events using elementary kinetic steps makes indirect response models (IDR) appealing. Indirect response models have been widely used in pharmacokinetic/pharmacodynamic models simulating the physiological response of a system exposed to an external signal or perturbation [51]. Thus we propose to model the effect of LPS on the transcriptional response level using the basic principles of an Indirect Response Model (IDR) [52], [53].

Of critical importance in analyzing dynamic systems is the identification of the state space that characterizes the behavior (response) of the system. To address this challenge, we recently developed a computational methodology that allows us to capture an elementary set of responses that describe the trajectory of systemic inflammation in human blood leukocytes when exposed to endotoxin stimulus [24], [25]. Such responses are maximally affected by the endotoxin stimulus and include the pro-inflammatory response that consists of the early increased expression of cytokines and chemokines; the anti-inflammatory response which is assumed to serve as the immunoregulatory arm of the host defense system and ultimately the energetic response that involves the decreased expression of genes that participate in cellular bio-energetic processes. We integrated these responses into a mathematical model using the basic principles of an Indirect Response Model (IDR) that bridges the extracellular signal (LPS) with the downstream activation of the major transcriptional responses. The model consists of eight (8) variables that include: (i) the inflammatory instigator (LPS), (ii) the endotoxin signaling free protein receptor (R, TLR4), (iii) the mRNA of TLR4 (mRNA,R), (iv) the formed complex (LPSR), (v) the active signaling complex (DR^*^) and the essential transcriptional responses (vi) pro-inflammation (P), (vii) anti-inflammation (A) and (viii) the energetic response (E). The mathematical representation of this model is succinctly presented in Eq. (1) as it follows:
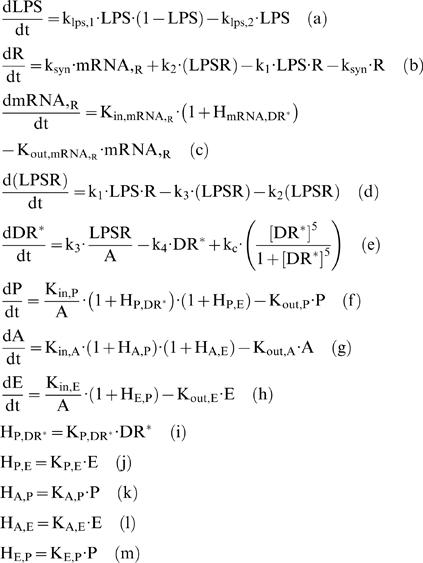
(1)


A detailed description of the structure and the mathematical representation of the aforementioned model is discussed in the original analysis [26]. Meanwhile the appropriateness of such a model was evaluated in a series of biologically relevant scenarios and involve: (i) a self-limited inflammatory response where the inflammatory stimulus is eliminated successfully, (ii) a persistent infectious response where the inflammatory instigator is not cleared from the system accounting for an aberrant inflammatory response and finally (iii) a persistent non-infectious response that can be elicited in response to an overload of the pathogen-derived endotoxin. In addition to this, the potential of the model was demonstrated by evaluating rapid endotoxin tolerance as well as potentiating effects.

### Developing an NFkB dependent indirect response model of inflammation

In order to introduce a finer level of detail in our computational model of inflammation we wish to deconvolute and interpret mechanistically the combined signal DR*. In the original model, DR* represent the event activating the transcription of the proinflammatory response (P) which in turn initiates the inflammatory response. As such, DR* is the signal activating, i.e., transcriptionally regulating, the expression of the pro-inflammatory genes. Thus, the mechanistic equivalent of DR* would be the signaling cascade that activates pro-inflammatory transcription factors controlling the expression of the pro-inflammatory genes. Although a large family of transcription factors is known to be involved in inflammation, we focus on a particular family, NFkB, for two reasons. First, the nuclear factor kB family is known to be a major player in the inflammatory response [54] and as such it has been widely studied as a major contributor. Second, the fact the NFkB plays an important role has led to the development of numerous, independent, modeling approaches in order to quantify the expected response of its signaling cascade [33]. Therefore, we introduce the NFkB signal transduction cascade as the prototypical module for initiating and controlling the expression of pro-inflammatory genes.

Numerous signaling molecules and reactions participate in the NFkB signaling pathway [33]. However, sensitivity analysis [55] demonstrated that the activity of NFkB is maximally modulated by a reduced set of basis signaling molecules (IKK, IKBa and NFkB). As such [56] proposed a minimal model of NFkB that accounts for the propensity of oscillations in the dynamic behavior of NF-kB activity. However, instead of simulating the kinase activity as a constant parameter and incorporating saturation degradation rates as discussed in [56], we propose to model IKK as a transient signal. Qualitatively, the dynamic IKK activity corresponds to its intracellular concentration and it serves as the “input signal” for the subsequent activation of NF-kB signaling module, Eq. (2).
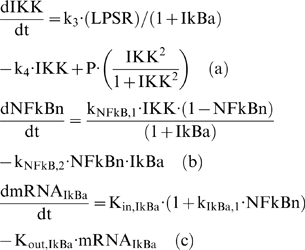
(2)


Thus the cellular surface complex (LPSR) induces the activation of kinase activity (IKK) with a rate k_3_, while being eliminated with a rate k_4_, (2a). The non-linear function of Hill-type, is an essential functional form in order to achieve a bistability response in the dynamics of the probed system [10], [57], [58], [59]. In chronic inflammatory diseases several cytokines might be responsible for perpetuating and amplifying the inflammatory reaction through the critical node (IKK) [36]. Therefore, we simulate such an interaction by the presence of a positive feedback loop in (2a). The dynamics of nuclear concentration of NF-kB are modeled in (2b) assuming NFkB_n_ as a percentage of its total cytoplasmic concentration. Therefore, the term (1-NFkB_n_) denotes the available free cytoplasmic concentration of NF-kB and in this study the nuclear concentration (NFkB_n_) and nuclear activity are used interchangeably. The import rate of cytoplasmic NF-kB into the nucleus depends on the availability of its free cytoplasmic concentration (1-NFkB_n_) stimulated by the kinase activity (IKK). However, its degradation rate depends on the presence of its primary inhibitor (IkBa) as the latter retrieves nuclear concentrations of NFkB by forming an inactive complex in the cytoplasmic region [60]. The dynamics of the gene transcript of IKBa (mRNA,_IKBa_), (2c), are characterized by a zero order production rate (K_in,IkBa_) and a first order degradation rate (K_out,IkBa_) which is stimulated by NFkB [36].

The protein inhibitor IkBa, as seen in Eq. (3), is the product of translation of its gene transcript (mRNA,_IkBa_) and it degrades at a rate k_I,2_ which is stimulated by the kinase activity (IKK). Based on the premise that IkB_a_ forms a complex with the available cytoplasmic NF-kB mathematically we expressed is as the product (1-NFkBn) IkBa. From the modeling point of view, in order to achieve a zero steady state for the protein inhibitor IkBa we need the additional negative term −k_I,1_.
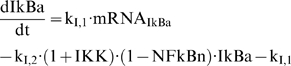
(3)


The dynamics of the gene transcript of the endotoxin signaling receptor (mRNA,_R_) are described by a zero order production rate (K_in,mRNA,R_) and a first order degradation rate (K_out,mRNA,R_), (4a).
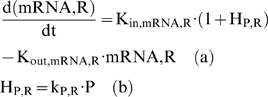
(4)


We test the hypothesis that the pro-inflammatory signaling indirectly stimulates the transcriptional activation of endotoxin receptor (TLR4); which quantitatively is expressed by the linear function (H_P,R_), (4b). Recently, there is research effort to elucidate the unknown mechanism that drives the regulation of TLR4 expression [61] and research findings [62] support the potential role of pro-inflammatory cytokines to up-regulate the TLR expression.

At the transcriptional response level, instead of assuming the active signaling complex, DR^*^ of Eq. (1f) to manifest the effect of LPS on the cellular response level, herein we assume that the nuclear activity of NF-kB (NFkB_n_) serves as the “active signal” that indirectly stimulates the production rate of the essential pro-inflammatory response (*P*), Eq. (5a).
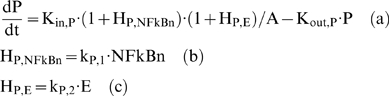
(5)


Mathematically the stimulation of the nuclear activity NFkB_n_ is expressed by the linear function (H_P,NFkBn_), (5b) and downstream of the pro-inflammatory response we preserve the structure of the elements that constitute the anti-inflammatory and the energetic response the same as shown in Eq. (1). For example, the energetic response variable will be responsible for more pronounced inflammation and therefore stimulates the pro-inflammatory response (H_P, E_), (5c). The anti-inflammatory signaling component is assumed to inhibit the production rate of the pro-inflammatory transcriptional signature, (5a). The transcriptional dynamics of anti-inflammation (A) and the energetic response (E) are modeled on the same manner as discussed in Eq. (1). The integrated NF-kB dependent indirect response model is presented in Eq. (6):
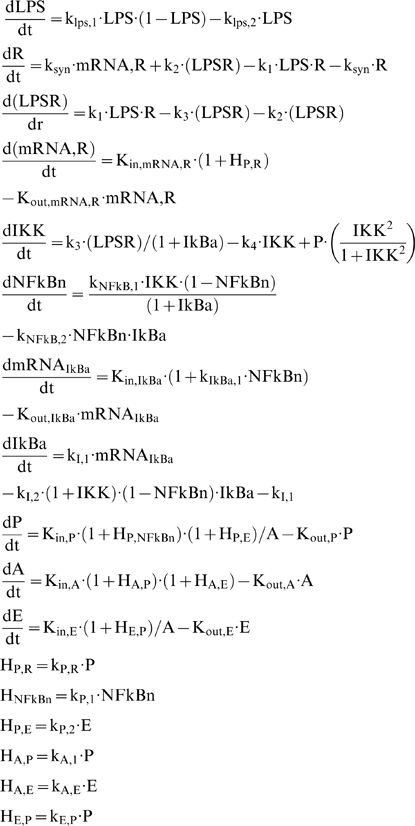
(6)


Two major differences exist between the model in Eq. (1) and the proposed NF-kB dependent indirect response model, Eq. (6). First, the “translation” of the active signaling complex (DR^*^) into biologically relevant signaling compartments; namely involving the activation of NFkB signaling module. Such a “translation” allows us to simulate the positive interaction between the pro-inflammation and the intracellular signaling (IKK). Second, in (1) the variable DR^*^ is assumed to be the convoluted signal that propagates the LPS signaling initiating the transcriptional synthesis of both the pro-inflammatory response (P) and the mRNA of TLR4 (mRNA,_R_). However, in model (6) the elucidation of DR^*^ to the NFkB activity limits the potential structure of the model. That is to say NFkB is a pro-inflammatory transcription factor and it is not involved in the transcriptional regulation of the gene that encodes for the protein TLR4. On the other hand, based on literature evidence we support the potential role of pro-inflammatory signaling in mediating the transcriptional machinery of TLR4 (mRNA,_R_) and we take it into consideration in the extended structure of the model, Eq. (6). The proposed NF-kB dependent indirect response model is schematically illustrated in Figure 1 shedding insight on the interactions of the elements that constitute the inflammatory response. It offers us “realistic” handles on evaluating the effectiveness of various intervention strategies that modulate the intrinsic dynamics of the system opening areas amenable to the design of effective treatment schedules [23]. In the present study we aim at exploring *in silico* the pharmacodynamic effect of particular immunomodulatory agents – corticosteroids - in modulating the progression of an unresolved inflammatory response

### Modeling Corticosteroid Interventions

The progression of a disease involves the perturbation in the intrinsic dynamics of a system from its homeostasis [14]. The presence of a disturbance (stimulus) initiates complex interaction of components at multiple scales (genetic, molecular, cellular level). The administration of a drug aims at modulating the progression of the disease by interfering with either individual molecules or signaling pathways. As such, we will explore means of modulating the activity of NFkB through the use of corticosteroids Developing mechanistic models of inflammation allows us to both characterize the non-linear inflammatory trajectory under various “what-if” scenarios and importantly to evaluate the effectiveness of drug-based treatment strategies that modulate the dynamics of the system. Integrating the cellular mechanism of drug action on disease progression models sheds insight on the better characterization of their pharmacodynamic effect against the disease status.

In the present study, we consider corticosteroids as the means for controlling (modulating) the inflammatory state. One of the key aspects is the integration of the opposing effect of two crucial signaling pathways: one associated with the transcriptional dynamics that are elicited in response to endotoxin stimulus (LPS) and one related to the genomic signaling of exogenous corticosteroids. Such a modeling approach allows us to explore the pharmacodynamic effect of corticosteroids against inflammation exploring various modes of action.

Significant prior research efforts have attempted to elucidate the mechanisms driving corticosteroid activity [63], [64], [65], [66], [67], [68], [69], [70] Such studies simulate the pharmacogenomic effect of glucocorticoids at the transcriptional level taking their mechanistic (signaling) action into account [71], [72] and mathematically is expressed by Eq. (7) [72].
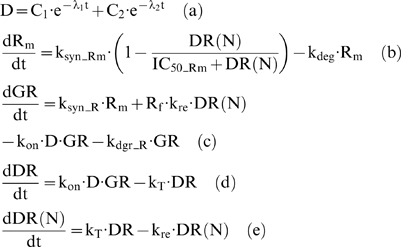
(7)


In essence the model as shown in Eq. (7) captures the essential steps of the cellular signaling of steroids which include: (i) the binding of the steroid drug (D) to its cytosolic receptor (GR), (ii) the subsequent formation of the drug-receptor complex (DR) (iii) the translocation of the cytosolic complex to the nucleus (DR(N)) which alters the transcriptional machinery activating or repressing numerous genes and finally (iv) the autoregulation of the gene transcript of the glucocorticoid receptor (R_m_).

The drug disposition is modeled via a bi-exponential kinetic model, (7a) and the plasma concentration of the drug (D) is mathematically expressed by a kinetic model with C_i_ and λ_i_ to be the coefficients of intercepts and slopes [69], [72]. The dynamics of the gene transcript of the corticosteroid drug (R_m_), (7b), are characterized by a zero production rate (k_syn_Rm_) and a first order degradation rate (k_deg_). The active drug-receptor complex (DR(N)) exerts an inhibitory effect towards the mRNA of the glucocorticoid receptor. The parameter IC_50_Rm_ denotes the concentration of the nuclear drug-receptor complex DR(N) at which the synthesis rate of the receptor drops at 50% of its baseline value. The dynamics of the free cytosolic receptor density, GR, is modeled in (7c) where k_syn_R_ is the synthesis rate of receptor that stems from its transcription, R_f_ is the fraction of the drug that is recycled, k_re_ is the parameter that shows the recycling of drug from the nucleus to the cytosol and k_on_ is a parameter associated with the drug-receptor binding. In addition to this, k_dgr_R_ is the degradation rate of the receptor (GR). The formed cytosolic complex (DR), (7d), depends upon the binding interaction k_on_ of the ligand (D) with its receptor (GR) and on its translocation rate k_T_ to the nucleus. Therefore, the translocation of the drug-receptor complex to the nucleus accounts for the nuclear receptor complex DR(N), (7e), which is the active complex that mediates the transcriptional induction of various genes.

Effectively, in [52] the model in Eq. (7) simulates in rat liver the effect of plasma concentration of a corticosteroid drug after a single intravenous administration of 50 mg/kg. The model parameters are estimated based on available experimental data and the qualitative structure of the integrated inflammatory model with the active corticosteroid intervention envelope is presented in Figure 6. We observe that the interaction of the corticosteroid drug (D) with its receptor (GR) mediates the activation of the nuclear drug-receptor complex (DR(N)). This complex serves as the “active signal” that induces transcriptional alterations suppressing the mRNA of the glucocorticoid receptor (R_m_) which drives downstream the reduced cytosolic receptor density.

Given, therefore, a quantification of the dynamics of corticosteroids and putative modes of action of CS in regulating the activity of NFkB [73], [74] we test the hypothesis that corticosteroids exert their immunosuppressive effect by enhancing the transcriptional synthesis of NF-kB's inhibitor IkBa (mRNA,_IkBa_). Such a hypothesis does not imply that corticosteroids exert their anti-inflammatory mechanisms via only this mechanism. It has become increasingly evident [30] that corticosteroids manifest their anti-inflammatory properties by various mechanisms that involve (i) either up-regulation of critical anti-inflammatory proteins, i.e. IkBa, IL-10; (ii) or increased expression of an inhibitor to phospholipase A_2_ (annexin I) which subsequently leads to reduced formation of both arachidonic acid and platelet-activating factor as well as (ii) a disruption of the basal transcriptional machinery that inhibits the transcriptional activity of NFkB. In this study, due to our inability to model all the mediators that may be affected by corticosteroids we opt to simulate the effect manifested by exogenous corticosteroids performing systematic perturbations on the primary inhibitor of NFkB, i.e. IkB, as shown in Eq. (8).
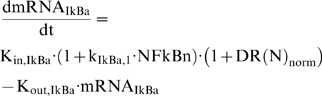
(8)where DR(N)_norm_ represents the normalized DR(N) signal that numerically ranges between (0,1) for a given drug dose. The reason for such normalization stems from the fact that the aim of this study is to provide a qualitative understanding about how the dynamics of a host undergoing an inflammatory response are modulated due to the corticosteroid intervention envelope.
